# Supplementary material for: Toward a 3D model of phyllotaxis based on a biochemically plausible auxin-transport mechanism
Source: PLoS Comput Biol. 2019 Apr 18;15(4):e1006896. doi: 10.1371/journal.pcbi.1006896 (PMC6490938; doi:10.1371/journal.pcbi.1006896)
Supplement: S1 Appendix — (PDF) [file pcbi.1006896.s001.pdf]

Towards a 3D model of phyllotaxis based on a biochemically plausible auxin-transport mechanism
Supporting Information

Félix P. Hartmann[1,2], Pierre Barbier de Reuille[1], Cris Kuhlemeier[1,*]

[1] Institute of Plant Sciences, University of Bern, 3013 Bern, Switzerland

[2] Université Clermont Auvergne, INRA, PIAF, F-63000 Clermont–Ferrand, France

[*] cris.kuhlemeier@ips.unibe.ch

1 Equations describing the model

Most differential equations for our model can be derived from the Petri net in Figure 1 (main text). We detail here all of them, using a graph describing how discrete elements of tissue are connected to each other (Figure S1).

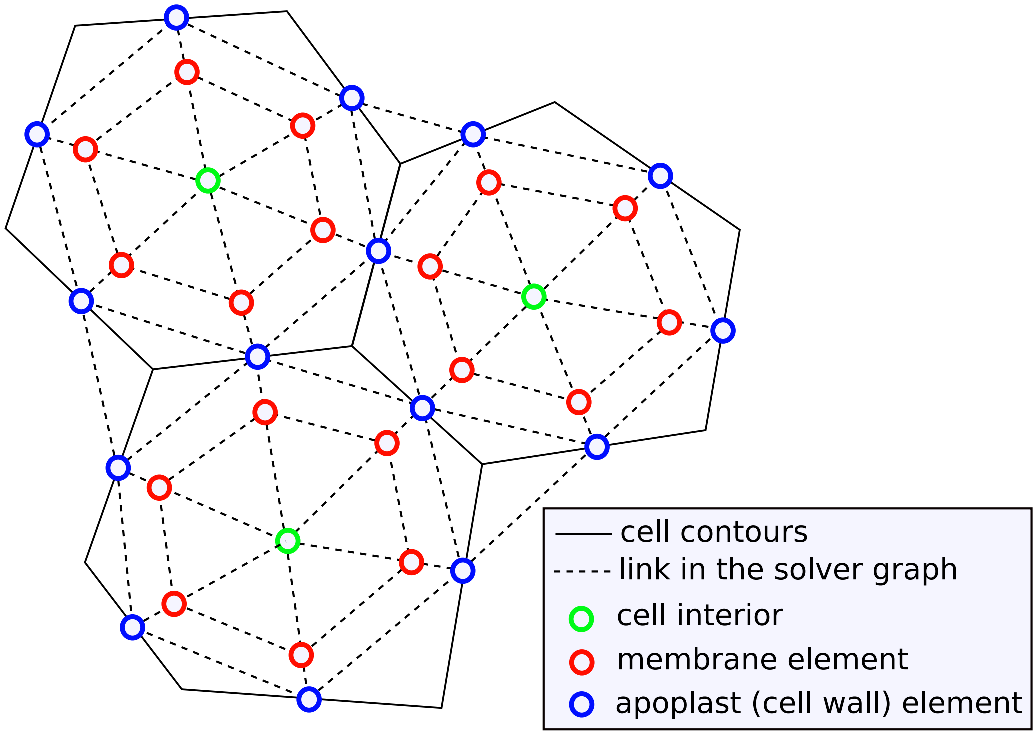

Figure S1: General structure of the graph used for solving the set of differential equation. Simplified representation in two dimensions with three cells. Each node (colored circles) represents a discrete element of tissue and is associated with numerical variables (local concentration of chemical species). Two nodes are connected by a link if fluxes of chemical species potentially exist between them. A cell interior is connected to all the membrane elements forming the membrane of the cell. A membrane element is connected to its associated apoplast element, to neighboring membrane elements and to a cell interior. An apoplast element is connected to its two associated membrane elements and to neighboring apoplast elements.

Let us first consider a cell interior c (a green node on the graph). It is associated with an auxin concentration $[A_c]$. The change $[A_c]$ is given by

$$\frac{\mathrm{d}\,[A_c]}{\mathrm{d}t} = \underbrace{\sigma_a - \mu_a\,[A_c]}_{\text{synthesis and decay}} + \sum_{\substack{\text{Membrane}[m]\\ \text{Connected}[c,m]}} \frac{\text{Area}\,[m]}{\text{Volume}\,[c]} \left(\underbrace{\nu_{apin}\,[APIN_m]\,[AAUX_m] + T_{in2}\,[AAUX_m] - T_{out1}\,[A_c]\,[PIN_m]}_{\text{complex formation and dissociation}} \right),$$

where the summation symbol

$$\sum_{\substack{\text{Membrane}[m]\\ \text{Connected}[c,m]}}$$

reads as "sum over all membrane elements m (red nodes) such that m is connected to c". "Volume $[c]$" is the volume of the cell c and "Area $[m]$" is the area of the membrane element m. $[PIN_m]$, $[APIN_m]$ and $[AAUX_m]$ are respectively the surface concentrations of PIN, APIN and AAUX molecules on the membrane element m. Parameters are explained in the main text and in Table S1.

The change in PIN concentration in c, $[PIN_c]$, is given by

$$\frac{\mathrm{d}\,[PIN_c]}{\mathrm{d}t} = \sum_{\substack{\text{Membrane}[m]\\ \text{Connected}[c,m]}} \frac{\text{Area}\,[m]}{\text{Volume}\,[c]} \left(\underbrace{\mu_p\,[PIN_m] - \sigma_p\,[PIN_c] - \sigma_{apin}\,[PIN_c]\,[APIN_m]^2 - \sigma_{aaux}\,[PIN_c]\,[AAUX_m]^2}_{\text{endocytosis - exocytosis}} \right) + \underbrace{\frac{\rho_p\,[A_c]}{1+\kappa_{PIN}\,[PIN_c]}}_{\text{auxin-dependent synthesis}} - \underbrace{\mu_{p\star}\,[PIN_c]}_{\text{decay}}. \quad (1)$$

Let us now consider a membrane element m (a red node on the graph). The change in PIN surface concentration in m,$[PIN_m]$, is given by

$$\frac{\mathrm{d}\,[PIN_m]}{\mathrm{d}t} = \underbrace{\sigma_p\,[PIN_c] + \sigma_{apin}\,[PIN_c]\,[APIN_m]^2 + \sigma_{aaux}\,[PIN_c]\,[AAUX_m]^2 - \mu_p\,[PIN_m]}_{\text{exocytosis-endocytosis}} \underbrace{+\nu_{apin}\,[APIN_m]\,[AAUX_m] + T_{out2}\,[APIN_m] - T_{out1}\,[PIN_m]\,[A_c]}_{\text{complex formation and dissociation}}, \quad (2)$$

where $[PIN_c]$ and $[A_c]$ are, respectively, the PIN and auxin concentrations in the unique cell interior c connected to m.

Changes in APIN and AAUX surface concentrations are given by

$$\frac{\mathrm{d}\,[APIN_m]}{\mathrm{d}t} = T_{out1}\,[PIN_m]\,[A_c] - \nu_{apin}\,[APIN_m]\,[AAUX_m] - T_{out2}\,[APIN_m]\,,$$

$$\frac{\mathrm{d}\,[AAUX_m]}{\mathrm{d}t} = T_{in1}\,[AUX]\,[A_a] - T_{in2}\,[AAUX_m]\,,$$

where $[A_a]$ is the auxin concentration in the unique apoplast element a (a blue node on the graph) connected to m, and $[AUX]$ is the (fixed) concentration of AUX/LAX importer.

Finally, let us consider an apoplast element a. The change in auxin concentration in a is given by

$$\frac{\mathrm{d}\left[A_a\right]}{\mathrm{d}t} = \frac{1}{\text{Volume}\left[a\right]} \sum_{\substack{\text{Apoplast}\left[a'\right]\\ \text{Connected}\left[a,a'\right]}} \underbrace{\text{Area}\left[a,a'\right] D_a \left(\left[A_{a'}\right] - \left[A_a\right]\right)}_{\text{diffusion in the apoplast}}$$

$$+ \sum_{\substack{\text{Membrane}[m]\\ \text{Connected}[m,a]}} \frac{\text{Area}\left[m\right]}{\text{Volume}\left[a\right]} \left(\underbrace{T_{out2}\left[APIN_m\right] - T_{in1}\left[AUX\right]\left[A_a\right]}_{\text{fluxes through membranes}} \right),$$

where the first sum runs over all apoplast elements connected to a, and the second one runs over the two membrane elements connected to the apoplast element a. "Area $[a, a']$" is the area of the border between apoplast elements a and a'.

The rate of APIN dissociation by AAUX, ν_{apin}, takes different values ν_{apin}^{low} and ν_{apin}^{high} depending on whether auxin cell concentration is, respectively, below or above a threshold a_{th}. The transitions between the two values is smooth and follows a steep logistic function:

$$\nu_{apin}\left(\left[A_c\right]\right) = \nu_{apin}^{\text{low}} + \frac{\nu_{apin}^{\text{high}} - \nu_{apin}^{\text{low}}}{1 + e^{-k\left(\left[A_c\right] - a_{th}\right)}}.$$

k is the steepness of the curve and is set to 25.

2 Lateral PIN diffusion between neighbor membrane elements

Lateral PIN diffusion is formally introduced as an additional term in Eq. 2:

$$\frac{1}{\text{Area}\left[m\right]} \sum_{\substack{\text{Membrane}\left[m'\right]\\ \text{Connected}\left[m,m'\right]}} \text{Length}\left[m,m'\right] D_p \left(\left[PIN\right]_{m'} - \left[PIN\right]_m\right),$$

where "Length $[m, m']$" is the length of the border between membrane elements m and m'.

3 Vein attraction factor (VAF)

The vein attraction factor polarizes the tissue toward the sinks. It is unloaded at a fixed rate by sink cells into the neighboring apoplast. The change in VAF concentration in an apoplast element a neighboring a sink cell is given by

$$\frac{\mathrm{d}\left[VAF_a\right]}{\mathrm{d}t} = \frac{\text{Area}\left[m\right]}{\text{Volume}\left[a\right]} \rho_{VAF},$$

where "Area $[m]$" is the area of the membrane element of the sink cell in contact with a.

VAF in the apoplast can bind to and unbind from a membrane element following the equation

$$\frac{\mathrm{d}\left[VAF_m\right]}{\mathrm{d}t} = \underbrace{k_b\left[VAF_a\right]}_{\text{binding}} - \underbrace{k_u\left[VAF_m\right]}_{\text{unbinding}}.$$

VAF diffused within the apoplast. The full equation (without neighboring sink) for the change in VAF concentration in an apoplast element is

$$\frac{\mathrm{d}\left[VAF_a\right]}{\mathrm{d}t} = \frac{1}{\text{Volume}\left[a\right]} \sum_{\substack{\text{Apoplast}\left[a'\right]\\ \text{Connected}\left[a,a'\right]}} \underbrace{\text{Area}\left[a,a'\right] D_{VAF} \left(\left[VAF_{a'}\right] - \left[VAF_a\right]\right)}_{\text{diffusion in the apoplast}}$$

$$+ \sum_{\substack{\text{Membrane}[m]\\ \text{Connected}[m,a]}} \frac{\text{Area}\left[m\right]}{\text{Volume}\left[a\right]} \left(\underbrace{k_u\left[VAF_m\right] - k_b\left[VAF_a\right]}_{\text{binding/unbinding}} \right) - \underbrace{\mu_{VAF}\left[VAF_a\right]}_{\text{decay}}.$$

When VAF molecules are bound to a membrane element, they locally increase the feedback of auxin efflux on PIN exocytosis toward this element. More precisely, the exocytosis rate σ_{apin} in Eqs. 1 and 2 is replaced with an effective rate $\sigma_{apin}^{\text{eff}}$ which exponentially increases with the local concentration of membrane-bound VAF:

$$\sigma_{apin}^{\text{eff}} = \sigma_{apin} b_{VAF}^{[VAF_m]}.$$

See figure S2 for a complete Petri net of the biochemical reactions including VAF.

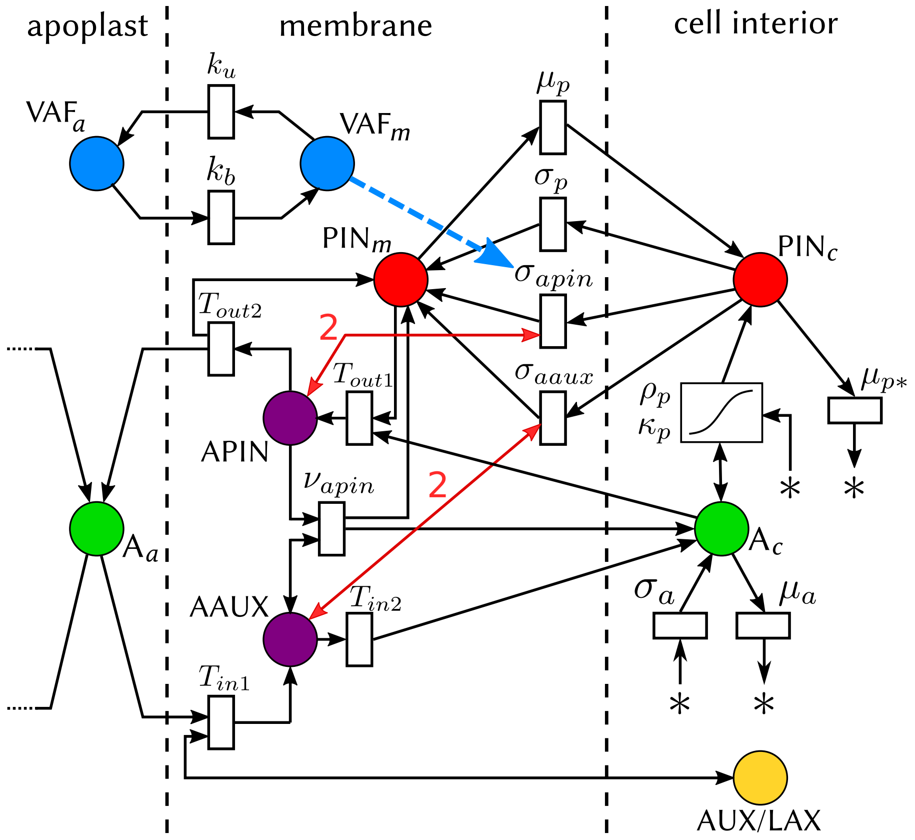

Figure S2: Petri net representation including binding and unbinding of VAF. The blue dashed arrow represents the influence of membrane-bound VAF on σ_{pin}.

4 Parameters used in simulations

Table S1: List of parameters used in simulations, with their values.

Parameter	Symbol	Value	Dimension[a]
Apoplast width		0.1	L
Apoplast auxin diffusion	D_a	0.01	L T^{-1}
PIN lateral diffusion	D_p	0.05	L T^{-1}
AUX/LAX concentration	$[AUX]$	1	M L^{-2}
Formation of AAUX	T_{in1}	1	L^3 M^{-1} T^{-1}
Formation of APIN	T_{out1}	1	L^3 M^{-1} T^{-1}
Influx by AAUX	T_{in2}	1	T^{-1}
Efflux by APIN	T_{out2}	2	T^{-1}
Auxin biosynthesis			
- in L1	σ_a^{L1}	0.1	M L^{-3} T^{-1}
- in inner layers	$\sigma_a^{\mathrm{inner}}$	0	M L^{-3} T^{-1}
Auxin decay			
- in sink cells	μ_a^{sink}	5	T^{-1}
- in non-sink cells	μ_a	0.05	T^{-1}
Auxin-dependent PIN biosynthesis	ρ_p	0.5	T^{-1}
PIN biosynthesis saturation	κ_{PIN}	1	L^3 M^{-1}
PIN decay	μ_p	0.05	T^{-1}
PIN exocytosis			
- constitutive	σ_p	0.05	L T^{-1}
- by APIN	σ_{apin}	4	L^5 T^{-1} M^{-2}
- by AAUX	σ_{aaux}	0.05	L T^{-1}
PIN endocytosis	μ_p	2	T^{-1}
threshold auxin concentration	a_{th}	1.65	M L^{-3}
APIN dissociation by AAUX			
- if $[A_c] < a_{th}$	$\nu_{apin}^{\mathrm{low}}$	0.3	L^2 T^{-1} M^{-1}
- if $[A_c] > a_{th}$	$\nu_{apin}^{\mathrm{high}}$	15	L^2 T^{-1} M^{-1}
ν_{apin} transition steepness	k	25	L^3 M^{-1}
VAF release at sink surface	ρ_{VAF}	0.1	M L^{-2} T^{-1}
VAF decay	μ_{VAF}	0.01	T^{-1}
VAF binding constant	k_b	0.1	L T^{-1}
VAF unbinding constant	k_u	0.1	T^{-1}
VAF diffusion	D_{VAF}	0.05	L T^{-1}
VAF effect on PIN exocytosis	b_{VAF}	3	without dimension

[a] Dimensions are specified using L for length, M for mass, and T for time. Actually, lengths are in µm.

5 Correlations between parameters

Parallel simulations with various parameter values have been run using the original model by Cieslak et al. (figure 9c), on a regular square grid. We only retained successful simulations, i,e. simulations in which convergence and canalization occurred properly. Then, we looked at how the parameters values were distributed over the 'population' of successful simulations. To that end, we plotted the parameters pair-wise (figure S3). In any subplot of figure S3. each point is a successful simulation. In many subplots, points are close to a straight line, which means that the two associated parameters are linearly correlated. Correlations are represented in figure S4 as a graph.

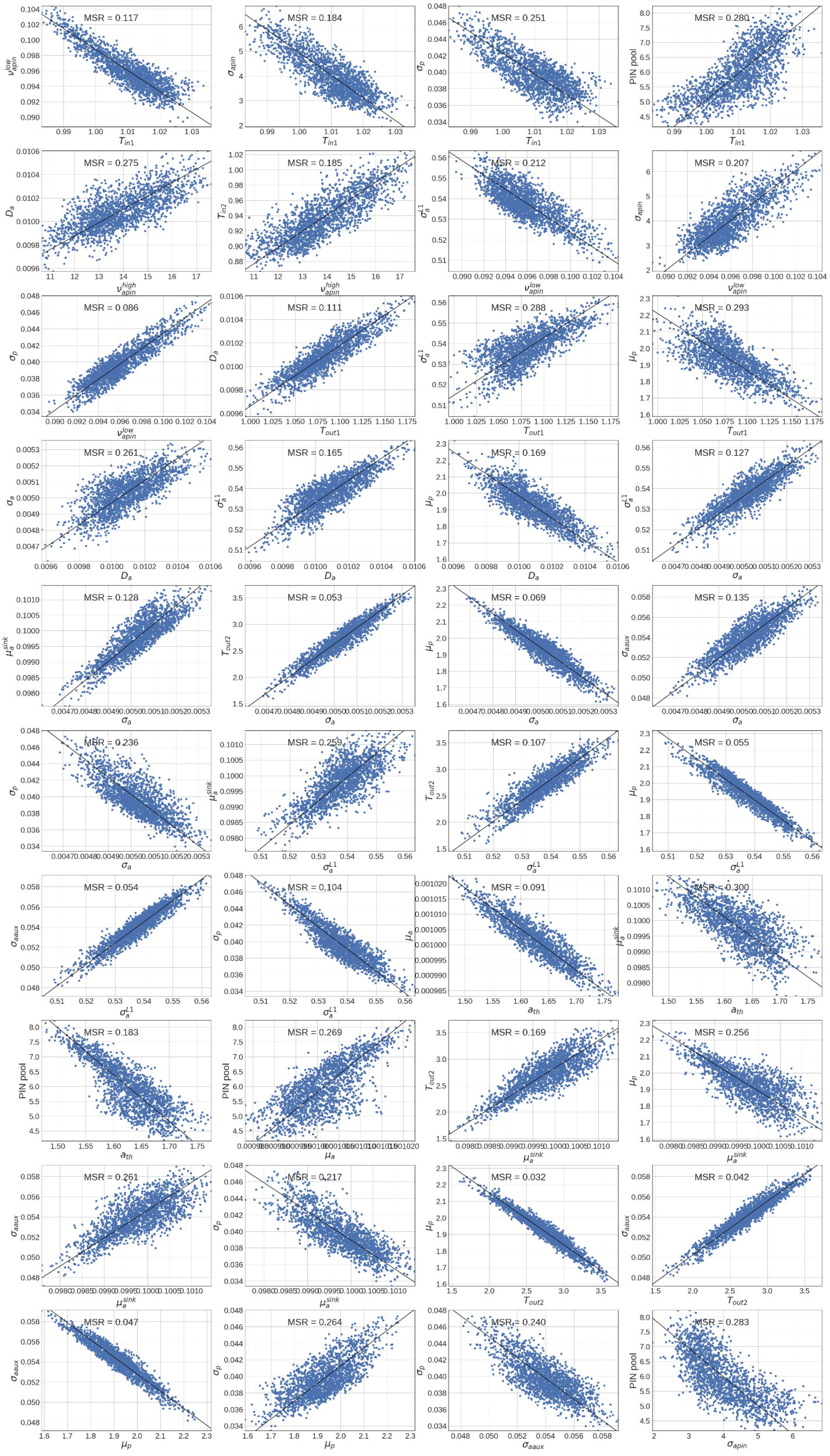

Figure S3: **Pair correlations between parameters.** Each point represent a successful simulation. For each pair of parameters, a linear regression has been performed. 'MSR' stands for *mean-square of residuals*. The closer to zero is the MSR, the stronger is the correlations between the pair of parameters.

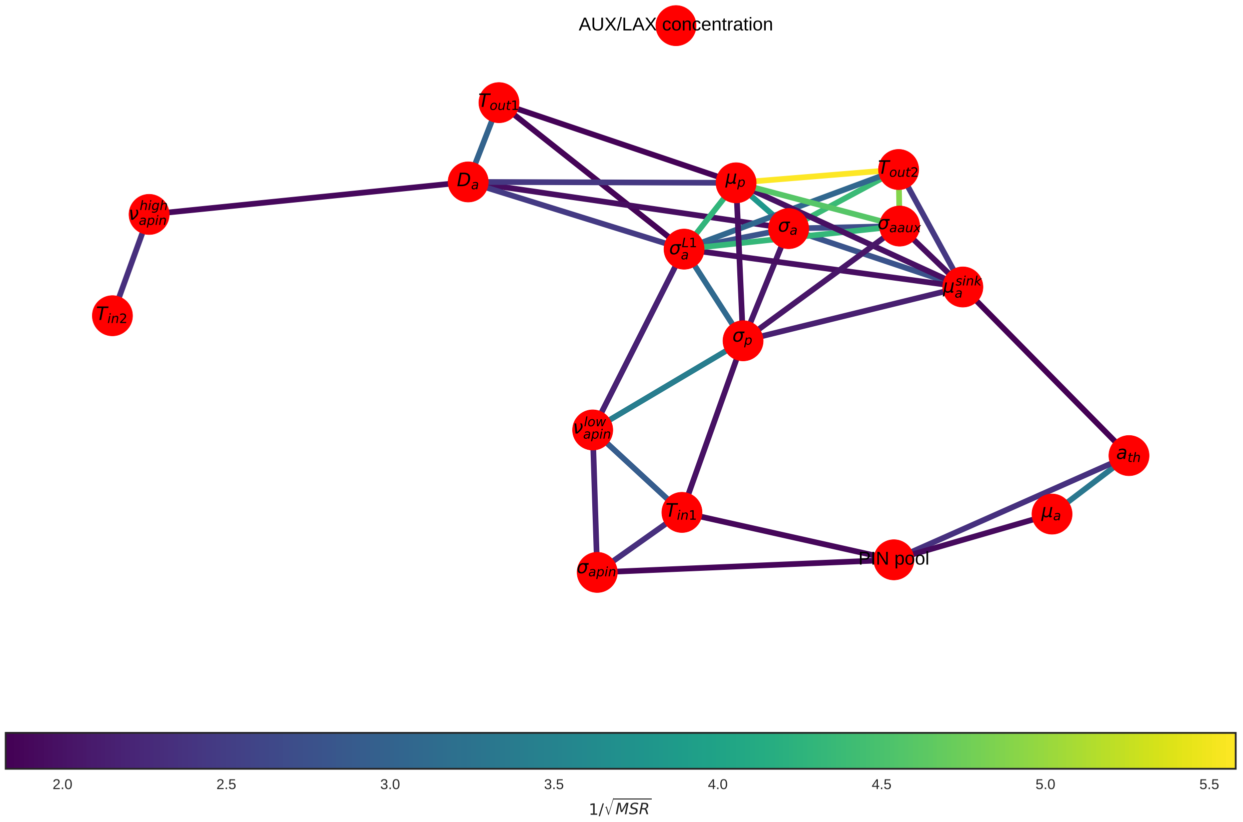

Figure S4: Graph of correlations between parameters. The nodes are linked if and only if the associated parameters are linearly correlated. The link color shows the strength of the correlation (a brighter color means a stronger correlation). The concentration of AUX/LAX protein carriers is not correlated with any other parameter.

Two parameters were particularly sensitive: The threshold auxin concentration a_{th} and the transition steepness k. Therefore, we assessed the sensitivity of the 3D model to these two parameters individually. We found that the threshold auxin concentration a_{th} was limited to the quite narrow range $[1.65, 1.75]$. For lower values of a_{th}, we could not observe canalization. For higher values of a_{th}, venation was less efficient and we had to increase the VAF production rate to ensure connection to all sinks. Regarding the transition steepness k, we found a minimum value (24) for venation to occur. For very high values of steepness, numerical instabilities can arise, but this is not an essential feature of the model, just a limitation of our solver.
